# Intra-operative hyperoxia and the risk of delirium in elderly patients after cardiac surgery

**DOI:** 10.18632/aging.103058

**Published:** 2020-04-19

**Authors:** Anna Kupiec, Barbara Adamik, Katarzyna Forkasiewicz-Gardynik, Waldemar Goździk

**Affiliations:** 1 Department of Anesthesiology and Intensive Therapy, Wroclaw Medical University, Wroclaw 50-556, Poland

**Keywords:** hyperoxia, elderly, delirium, cardiac surgery, cardiopulmonary bypass

## Abstract

Delirium is a common complication after cardiac surgery. The aim of our study was to determine the impact of hyperoxia episodes occurring during cardiopulmonary bypass (CBP) on the rate of delirium episodes in the postoperative period. 93 patients, aged ≥ 65, who underwent elective cardiac surgery (CBP <90 minutes) were enrolled. The occurrence of delirium episodes was examined every 12 hours for three days after surgery. Eleven patients (11.8%) developed postoperative delirium (POD (+)) and 83 did not (POD (-)). More incidences of severe hyperoxia (PaO2 ≥ 26.6kPa) during CBP were observed in the POD (+) group: 64% had ≥ 2 episodes of hyperoxia, 27% ≥ 3, and 18% ≥ 4, while in the POD (-) group: 42%, 13% and 1%, respectively (P=0.02). Patients in the POD (+) group had a higher maximum PaO_2_ during CBP than the POD (-) group (37 ± 5.8 vs 31.6 ± 6.6 kPa; P=0.01) and a higher mean PaO_2_ (30.1 ± 4.5 vs 26.1 ± 5.6 kPa; P=0.01). The optimal maximum PaO_2_ cut-off point for the occurrence of delirium was 33.2 kPa (AUC 0.72, P=0.001, sensitivity 75%, specificity 38%). We conclude that CBP hyperoxia episodes may be a risk factor associated with the occurrence of postoperative delirium.

## INTRODUCTION

Delirium is one of the most common complications after cardiac surgery. The incidence rates range from 11% up to 52% [1]. The presence of delirium after cardiac surgery is associated with a higher mortality rate, increased length of Intensive Unit (ICU) stay [2], higher hospitalization costs [3] and a risk of readmission [4]. Furthermore, the occurrence of delirium during the postoperative period correlates with the development of long-term consequences after surgery such as cognitive decline and functional impairment, which in turn results in the deterioration of the patient’s quality of life [5].

The etiology of delirium is multifactorial and it is always a combination of predisposing and precipitating risk factors [6]. Several pathophysiological mechanisms that contribute to the development of postoperative delirium have been presented, such as inflammation, disturbances in cerebral autoregulation, aortic plaque disruption, microemboli, Alzheimer-like cerebral pathology, and the neurotoxicity of anesthetics [7].

The role of low oxygen concentration in the development of delirium has been previously described. Severe hypoxia during CPB is one of the risk factors for postoperative delirium [8] and intraoperative cerebral oxygen desaturation and disturbances in the oxygen supply-demand balance may contribute to acute brain failure [7, 9]. One of the main tasks for the anesthesiologist during cardiac surgery is to prevent cellular hypoxia and high concentrations of oxygen are often administered. As a consequence, hyperoxia episodes may occur that are as dangerous to the patient as the occurrence of hypoxia. Increasing amounts of data are demonstrating worse clinical outcomes related to hyperoxia. The presence of hyperoxia episodes were reported in over 43% of emergency department patients [10], 36% of patients after cardiac arrest [11] and up to 70% of patients hospitalized in the ICU [12].

The role of hyperoxia as a risk factor for the development of delirium in patients undergoing cardiac surgery with cardiopulmonary bypass (CPB) has not yet been determined. The aim of this study was to determine the impact of hyperoxia episodes occurring during CBP on the incidence of delirium in the immediate postoperative period.

## RESULTS

The characteristics of the study population are shown in Table 1. Out of 93 patients included in the study, 11 (11.8%) developed postoperative delirium (group POD (+)) and 82 did not (group POD (-)). There were no significant differences between groups in age, gender, or in factors related to the patient’s clinical condition assessed with the Euroscore II and Charlson Comorbidity Index. There was no statistical difference between the study groups in the incidence of comorbidities such as recent myocardial infarction, lung disease, peripheral vascular disease, history of atrial fibrillation, diabetes mellitus or cerebral vascular disease (Table 1). There were no significant differences in the incidence of postoperative complications such as bleeding, need for re-thoracotomy, need for red blood cell transfusions, or acute kidney injury. 2 patients (18%) in the POD (+) group and 5 patients (6%) in the POD (-) group received milrinone infusion (P=0.15). No one needed adrenaline infusion and noradrenaline infusion was given to 6 patients (54.5%) in the POD (+) group and 40 patients (48.7%) in the POD (–) group (P=0.71). The highest dose of noradrenaline was similar in both groups (P=0.65) (Table 2).

**Table 1:** Demographic characteristics of the study population.

	**POD (+)**	**POD (-)**	**p**
	**N=11 (11.8%)**	**N=82 (88.2%)**	
Gender, male [N, (%)]	6 (54.5%)	57 (69.5%)	.33
Age [years]	74 ± 6 (65 - 87)	71 ± 5 (65 - 83)	.13
BMI [kg/m^2^]	28 ± 3.2 (22.5 - 33.6)	28.5 ± 4.4 (20.8 - 38.2)	.87
BSA [m^2^]	1.86 ± 0.19 (1.54 - 2.23)	1.92 ± 0.19 (1.53 - 2.37)	.38
Euroscore II [%]	6.0 ± 9.6 (0.8 - 33.1)	2.4 ± 2 (0.5 - 9.4)	.38
Charlson Comorbidity Index	5.7 ± 2 (3 - 10)	4.7 ± 2 (2 - 10)	.15
CCS IV [N, (%)]	1 (9)	2 (2.4)	.24
NYHA III/ IV [N, (%)]	4 (36.3)	13 (15.8)	.09
Comorbidities [N, (%)]			
Recent myocardial infarction	3 (27.7)	19 (23.2)	.76
Lung disease	0 (0)	4 (4.9)	-
Peripheral vascular disease	1 (9)	10 (12.2)	.76
History of atrial fibrillation	2 (18.1)	11 (13.4)	.66
Diabetes mellitus	6 (54.5)	31 (37.8)	.28
Cerebrovascular disease	3 (27.2)	10 (12.2)	.17
Chronic kidney disease	3 (27.2)	16 (19.5)	.54
Ejection fraction [%]	54 ± 12 (25-65)	57 ± 8 (30-68)	.80
Mechanical ventilation time [min]	292 ± 177 (90 - 690)	231 ± 94 (95 - 555)	.31
ICU length of stay [days]	3 ± 1.6 (2 - 7)	3 ± 1.6 (2 - 9)	.68
Length of hospitalization [days]	10 ± 1.6 (8 - 13)	9.8 ± 2.7 (7 - 24)	.10

POD: Postoperative delirium; BMI: Body Mass Index; BSA: Body Surface Area; ICU: Intensive Care Unit; CCS: Canadian Cardiovascular Society Angina Grading Scale; NYHA: New York Heart Association Functional Classification; Data are presented as mean ± SD (minimum - maximum) or percentage.

**Table 2:** Surgical parameters.

	**POD (+)**	**POD (-)**	**p**
	**N=11**	**N=82**	
CBP time [min]	71.6 ± 12.3 (48 - 89)	70 ± 12.4 (37 - 90)	.75
AoX time [min]	39.4 ± 13.6 (20 - 68)	39.1 ± 11.7 (19 - 69)	.98
Cardiac Index on CBP [L/min/m2]	2.5 ± 0.3 (2.0 - 3.0)	2.5 ± 0.1 (2.1 - 2.9)	.69
Mean Hb level on CBP [g/dl]	9.3 ± 1.8 (7.2 - 12.8)	9.4 ± 1.0 (7.4 - 11.6)	.48
Type of surgery [N, (%)]			.52
Isolated CABG	9 (81.8)	67 (81.7)	
Isolated valve surgery	1 (9.0)	13 (15.8)	
Combined procedure	1 (9.0)	2 (2.4)	
Perioperative vasopressors [N, (%)]	6 (54.5)	40 (48.7)	.71
Noradrenaline, highest [μg/kg/min]	0.05 ± 0.08 (0 - 0.22)	0.03 ± 0.04 (0 – 0.18)	.65
Milrinone infusion [N, (%)]	2 (18)	5 (6)	.15
Perioperative RBC transfusion [ml]	305 ± 292 (0 – 840)	198 ± 347 (0 – 1960)	.15
Postoperative blood loss [ml]	365 ± 177 (200 – 750)	414 ±182 (150 – 1050)	.27
Acute kidney injury [N, (%)]	1 (9.0)	7 (8.5)	.95
Lactate [mmol/L]	1.02 ± 0.29 (0.75 – 1.8)	1.02 ± 0.35 (0.4 – 2.46)	.99
pH	7.36 ± 0.04 (7.3 – 7.41)	7.37 ± 0.04 (7.23 – 7.46*)*	.92
Electrolyte balance [mmol/L]			
Na^+^	138.4 ± 5.2 (125.2 – 145.6)	139.2 ± 2.1 (135.6 – 150.3)	.92
K^+^	5.1 ± 0.4 (4.5 – 5.8)	5.1 ± 0.4 (3.9 – 6.3)	.33
Ca^2+^	1.18 ± 0.08 (1.06 – 1.38)	1.17 ± 0.05 (1.06 – 1.36)	.77
Re-thoracotomy [N, (%)]	0	2 (2,5)	-

POD: Postoperative delirium; CBP: Cardiopulmonary Bypass; AoX time: aorta cross-clamp time; Hb: hemoglobin; RBC: Red Blood Cells; CABG: coronary artery bypass graft, Combined procedure: valve surgery + coronary artery bypass graft; Data are presented as mean ± SD (minimum - maximum) or percentage.

The mean ICU length of stay was 3 days in both groups. There were no significant differences between groups in factors related to the operation such as CBP time, aortic cross clamping time, and mean hemoglobin level and cardiac index during CBP (Table 2). There was no significant difference between groups in blood loss or red blood cell transfusions and detailed information is provided in Table 2. No patient was given steroids perioperatively. After terminating sedation all patients were extubated on the day of surgery and the mean mechanical ventilation time was 292 minutes in group POD (+) and 231 minutes in the POD (-) group (n.s.). The hospital survival rate was 100% in both groups.

### Hyperoxia and delirium incidences

The Confusion Assessment Method for the Intensive Care Unit (CAM ICU) conducted before surgery indicated that delirium was absent in all patients included in the study. 81% of all delirium episodes occurred on the first postoperative day, 18% on the second, and 18% on the third day. The cumulative number of delirium episodes was greater than 100% because some patients had delirium episodes diagnosed more than once during the study period.

**Figure 1:**
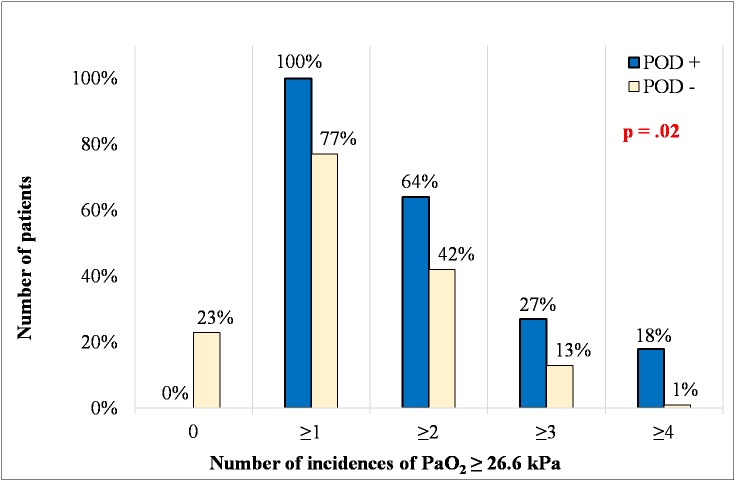
**Number of incidences of PaO_2_ ≥ 26.6 kPa in the studied groups.** The given p value represents the difference in the frequency of hyperoxia incidences for all data in figure 1. POD: postoperative delirium; PaO_2_: Partial pressure of oxygen.

Arterial blood gases were controlled every 20-30 minutes during CBP and a total of 256 of samples were analyzed. Severe hyperoxia defined as the highest PaO_2_ ≥ 26.6 kPa was recorded in 100% (11/11) of patients in the POD (+) group and in 78% (64/82) of patients in the POD (-) group. Mild hyperoxia defined as a PaO_2_ between 16 and 26.5 kPa was not observed in any patient in the POD (+) group but it was present in 21.9% (18/82) of the patients in the POD (-) group. During CBP, the incidence rate of severe hyperoxia was significantly higher in the POD (+) group than in the POD (-) group. The median number of episodes of severe hyperoxia was 2 (IQR 1-3) in the POD (+) group and 1 (IQR 1-2) in the POD (-) group. (P=0.02, Figure 1).

The value of the maximum PaO_2_ during CBP was significantly higher in the POD (+) group than in the POD (-) group (37 kPa vs 31.6 kPa; P=0.01). Similarly, the value of the mean PaO_2_ during CBP was significantly higher in the POD (+) group than in the POD (-) group (30.1 kPa vs. 26.1 kPa; P=0.01) (Table 3). There was no difference in the minimum PaO_2_ between groups (24.1 kPa vs. 21.2 kPa; P=0.06). Using the ROC curve, it can be concluded that the optimal maximum PaO_2_ cut-off point for the occurrence of delirium in the postoperative period was 33.2 kPa with an area under the curve 0.72 (P=0.001, sensitivity of 75%, specificity of 38%). Postoperative delirium was not associated with longer mechanical ventilation (292 min vs. 231 min, respectively, in the POD (+) and POD (-) group, P=0.31) or longer hospitalization after operation (10 vs. 9.8 days, respectively, in the POD (+) and POD (-) group, P=0.1).

**Table 3:** Results of data analysis.

	**POD (+)**	**POD (-)**	**p**
	**N=11**	**N=82**	
Mild hyperoxia (PaO_2_ 16-26.5 kPa) [N (%)]	0/11 (0.0)	18/82 (21.9)	.18
Severe hyperoxia (PaO_2_ ≥26.6 kPa) [N (%)]	11/11 (100.0)	64/82 (78.1)	.18
PaO_2_ max [kPa]	37 ± 5.8 (29.8 - 45)	31.6 ± 6.6 (19.8 – 52.6)	.01
PaO_2_ mean [kPa]	30.1 ± 4.5 (24.2 – 37.7)	26.1 ± 5.6 (15.3 – 41.6)	.01
PaO_2_ min [kPa]	24.1 ± 4.5 (18.2 – 33.8)	21.2 ± 5.9 (9.9 – 39.1)	.06

POD: Postoperative delirium; PaO_2_ max: maximum value of partial pressure of oxygen within CBP; PaO_2_ mean: mean value of partial pressure of oxygen within CBP; PaO_2_ min: minimal value of partial pressure of oxygen within CBP; CBP: Cardiopulmonary Bypass; Data are presented as mean ± SD (minimum; maximum).

## DISCUSSION

The study showed that despite the short time of CPB and without complications during surgery and during the ICU stay, up to 11.8% of patients experienced delirium in the period immediately after the surgery.

In the group of patients with delirium, the incidence rate of severe hyperoxia during CPB was significantly higher than in patients with an uneventful postoperative course. Thus, CBP hyperoxia episodes may be a risk factor associated with the occurrence of postoperative delirium.

Data on the negative consequences of hyperoxia are becoming more widely known. The results of previously published studies indicate that in a wide range of medical conditions such as myocardial injury, cardiac arrest, stroke, and respiratory failure requiring mechanical ventilation, hyperoxia is associated with increased morbidity and hospital mortality [11, 13, 14]. In our study, the occurrence of hyperoxia during surgery did not change mortality, and the survival rate in the hospital was 100%. However, this study group was a low risk population; all patients could be extubated on the day of surgery, without the need for a prolonged ICU stay.

The influence of hyperoxia on brain activity has been the subject of preclinical and clinical studies; however, the results of these studies are often contradictory. The results of preclinical studies indicate that prolonged hyperoxia induces a disturbance in the balance between the production of reactive oxygen species (ROS) and antioxidant defense [15, 16]. As a consequence, neuron apoptosis and cerebral tissue ROS increase, leading to persistent cerebral damage. In a clinical study investigating the effect of hyperoxia in patients with severe traumatic brain injury, a PaO_2_ above 19.9 kPa was shown to contribute to an increase in the glutamate level in brain tissue, suggesting secondary brain damage [17]. These results were not confirmed in a recently published retrospective analysis of over 24,000 patients after traumatic brain injury; hyperoxia > 39.8 kPa during the first 24 hours in the ICU, although present in 13% of patients, was not associated with a worse outcome [18]. In another study, Rincon et al. demonstrated the detrimental effect of hyperoxia in ventilated ICU patients after stroke; a PaO_2_ above 39.9 kPa was associated with higher in-hospital mortality and hyperoxia was even more harmful than hypoxia below 7.9 kPa [19]. These data underline the need for studies of controlled oxygenation in ventilated ICU patients.

To date, the impact of hyperoxia on the brain activity of patients undergoing cardiac surgery with CPB has been the subject of very few studies and this problem requires thorough analysis. The results of our study indicate a direct relationship between the high intraoperative PaO_2_ level and the occurrence of delirium in the immediate postoperative period in elderly patients. During CBP, severe arterial hyperoxia (PaO_2_ ≥ 26.6 kPa) was recorded in all patients who postoperatively developed delirium, and the incidence rate of severe hyperoxia recorded during CPB was significantly higher in patients with delirium than in those with an uneventful postoperative course.

In a study by Lopez et al., cerebral oxygenation was measured with oximetry monitors in cardiac surgery patients. The results indicated that prolonged cerebral hyperoxia during CBP was responsible for the occurrence of delirium following surgery in 30% of patients. What is more, the risk of developing delirium significantly increased when an episode of hypoxia was followed by prolonged hyperoxia [20]. In a recently published study by Thudium et al. a relationship was found between cerebral hyperperfusion and post cardiac surgery delirium. In the study the authors examined right middle cerebral artery blood flow during CBP using the transcranial Doppler [21]. In our study we did not plan to measure cerebral blood flow, but the cardiac index during CBP was similar in both groups, with and without postoperative delirium (mean CI=2,5 l/min/m^2^, P=0.69).

Although data on the negative effects of hyperoxia are increasing, toxic oxygen levels can still be found in the operating room. The safety of maintaining near-physiological levels of oxygenation during CPB was confirmed in a randomized clinical trial. It was demonstrated that the procedure of using an almost physiological level of oxygenation during CPB did not increase the level of lactate and troponin after surgery and did not increase the frequency of hypoxic episodes [22].

Delirium is always a result of many factors the majority of which are unpreventable. One important risk factor is prolonged CBP time. A detailed analysis of 1863 cardiac surgery procedures indicated that CPB time ≥90 minutes was considered to be prolonged and was associated with a longer hospital stay and a higher mortality rate [26]. In another study CPB ≥90 was associated with an increased release of the biomarkers of ischemia/reperfusion intestinal damage and endotoxemia [27]. Since we have no influence on CBP duration, careful attention to even the smallest details during surgery that may potentially have an impact on the postoperative course, are therefore, very important. Control and prevention of perioperative hyperoxia can be one such task. In our study, samples of blood-gases were controlled every 20-30 minutes and gas flow settings were corrected accordingly. The implementation of real-time PaO_2_ measuring devices can help reduce both hypoxia and hyperoxia episodes.

We are aware of our study limitations. The study was deliberately limited to elderly patients, a population at higher risk of postoperative delirium. We did not plan to analyze the long-term consequences of hyperoxia during CPB on brain injury. However, in a recently published retrospective analysis of 1018 patients undergoing cardiac surgery with CPB no association was found between hyperoxia >26.6 kPa or >39.9 kPa during surgery and neurocognitive dysfunction 6 weeks after surgery [23].

Additional research conducted on a larger population, with the assessment of cerebral oximetry and cerebral blood flow, and an examination of the immediate and long-term consequences of hyperoxia, will shed light on the question of the effects of hyperoxia on brain function after cardiac surgery. Future research in this area is needed and should also include the impact of hyperoxia on brain damage and the release of brain-specific biomarkers. Changes in biomarkers in patients experiencing hyperoxia episodes could be associated with cognitive decline and delirium after surgery.

Considering the lack of statistically significant differences between groups in the values of the other parameters studied in the perioperative period, it can be assumed with high probability that intraoperative hyperoxia may be the cause of postoperative delirium.

Delirium is always a result of many factors. As we have no influence on many of them, preventing delirium is always a difficult task. Since hyperoxia during CBP is an important risk factor for developing postoperative delirium, avoiding hyperoxia above 26.6 kPa during CBP, along with avoiding large variations in oxygen tension, seems an easy and safe measure to lower the risk of this complication.

## MATERIALS AND METHODS

This retrospective study was conducted at the 6-bed cardiosurgical ICU at the Department of Anesthesiology and Intensive Therapy, Wroclaw Medical University in Poland between December 2016 and March 2018. The Bioethics Committee of Wroclaw Medical University approved the study (permission no. 219/2016). Inclusion criteria were: age ≥ 65 years and elective on-pump cardiac surgery. Exclusion criteria were: CBP time ≥ 90 minutes, emergency procedure, a different type of anesthesia (TIVA, total intravenous anesthesia), or refusal to provide written informed consent.

### Delirium assessment

Each patient was examined for delirium before surgery (baseline) and then every 12 hours for three consecutive days, starting in the morning on the first day after surgery. The Confusion Assessment Method for the Intensive Care Unit (CAM ICU) was used for to identify the presence or absence of delirium. CAM ICU is a validated and commonly used score to assess delirium in ICU patients. With CAM ICU four features of delirium are assessed: 1) acute onset and a fluctuating course, 2) inattention, 3) disorganized thinking, and 4) an altered level of consciousness. To diagnose delirium, feature 1, 2, and either 3 or 4 should be recognized as positive. As a part of CAM ICU evaluation, the Richmond Agitation-Sedation Scale is used to assess altered levels of consciousness and fluctuations of mental status [24]. The score is recommended as a screening tool for postoperative delirium by the European Society of Anesthesiology [25]. All physicians involved in the study were trained on how to perform the CAM ICU delirium screening.

Every episode of hyperoxia that occurred during CPB was recorded; mild hyperoxia was defined as a value of the PaO_2_ between 16 and 26.5 kPa and severe hyperoxia as PaO_2_ ≥ 26.6 kPa. These cut-offs of hyperoxia are based on the results from previous studies [13]. Delirium is always a result of a combination of patient-related and operation-related factors. One important risk factor is prolonged CBP time. Detailed analysis of 1863 cardiac surgery procedures indicated that CPB time ≥90 minutes was considered to be prolonged and was associated with a longer hospital stay and a higher mortality rate [26]. In another study CPB ≥90 minutes was associated with an increased release of the biomarkers of ischemia-reperfusion intestinal damage and endotoxemia [27]. Therefore, to eliminate the impact of this factor and to gain better homogeneity of the sample group, we excluded patients with CBP time ≥ 90 minutes.

### Surgery

The induction of general anesthesia was achieved using sufentanil, propofol and rocuronium. During surgery the anesthesia was maintained using sevoflurane and a continuous infusion of sufentanil and rocuronium. Sevoflurane MAC was kept between 0.7 and 1.0. As a routine procedure, an open bypass circuit composed of uncoated polyvinylchloride tubing, a hard-shell venous reservoir, a hollow fiber membrane oxygenator (Medtronic Affinity, Medtronic, Inc. Minneapolis USA) with an integrated polyester arterial line filter of 40 μm pore size, and a roller pump with a nonpulsatile flow 2.4–2.5 l/min/m^2^ (Stockert S3, Sorin Group, Germany) was used in all patients. Anticoagulation was attained by administering 300 IU/kg of heparin to achieve an activated clotting time longer than 480 seconds. Perfusion pressure was kept at 60 to 80 mmHg. After aortic cross-clamping, cold blood cardioplegia Del Nido was used. Normothermia (37º C) was maintained during the entire procedure. Partial pressure of carbon dioxide (PaCO_2)_ was kept in a normal range between 4.6 – 5.9 kPa. During CBP, sevoflurane was administered to the extracorporeal circuit. Blood gas samples were taken by the perfusionist from the arterial line of the oxygenator every 20-30 minutes during CBP. According to our routine procedure, while on CBP, after every blood-gas result, gas flow settings were corrected on a regular basis: if the blood-gas result indicated a PaO_2_ below 20 kPa, the oxygen concentration was increased. Fresh gas flow was changed according to the PaCO_2_ blood gas result to keep the PaCO_2_ value within a normal range 4.6 – 5.9 kPa.

According to our routine procedure, mean arterial pressure (MAP) was measured continuously through an arterial catheter inserted to a radial or femoral artery. The lowest acceptable MAP was 65 mmHg. In the case of hypotension, patients were given a noradrenaline infusion.

Each patient was transferred to the ICU immediately after surgery. Sedation with propofol 0.5 - 1.0 mg/kg/h was continued until the patient was able to be weaned from the ventilator and extubated. Postoperative pain management was controlled using Paracetamol 1 g every 6 hours and Oxycodon 3-4 mg every 4 hours. Patients were extubated if they were hemodynamically stable with only a small amount of inotropic agents or catecholamines and with sufficient oxygenation.

### Statistical analysis

Data are expressed as mean values ± SD (minimum - maximum) or a number and percentage. The distribution of the variables was not normal based on a Shapiro–Wilk test. Therefore, statistical analysis of the data was performed using nonparametric tests. For continuous variables the Mann-Whitney U test was used to compare differences in maximal, minimal and mean PaO_2_ during CPB between two independent groups. Categorical variables were analyzed using a Yates’s chi-squared test. Receiver operating characteristics were analyzed to find the optimal maximum PaO_2_ cut-off point for the occurrence of delirium in the postoperative period and these results are presented as the area under the curve (AUC), standard error, and 95% confidence interval. The Mann–Whitney U test was used to compare differences between 2 independent groups. All statistical measurements were carried out with *Statistica* 13.1 (StatSoft Inc., Tulsa, USA). Statistical significance was determined as a P value less than *< 0.05.*
